# A trial of the iron‐chelator deferiprone in Alzheimer’s disease

**DOI:** 10.1002/alz.086330

**Published:** 2025-01-09

**Authors:** Scott Ayton, Ashley I. Bush, 3D trial investigators

**Affiliations:** ^1^ The Florey Institute of Neuroscience and Mental Health, The University of Melbourne, Australia, Melbourne, VIC Australia

## Abstract

**Background:**

Iron is vital for metabolism but can act as a catalyst for oxidative damage. Elevated brain iron, determined from biomarkers of iron (CSF ferritin and quantitative susceptibility mapping MRI) and from post‐mortem measurement of brain iron, has been associated with accelerated cognitive decline in multiple Alzheimer’s disease (AD) clinical, cohorts. These findings supported the hypothesis that treatment with the brain‐permeable iron chelator deferiprone may be associated clinical benefit in AD.

**Methods:**

A multicentre, phase II, double‐blind, randomized, placebo controlled clinical trial of deferiprone (15 mg/kg administered orally twice a day) was conducted in people with amyloid‐confirmed MCI and mild AD (MMSE≥20) over 12 months. The primary outcome was cognition, assessed at baseline, 6 months, 12 months using neuropsychological test battery of memory (3), executive function (3) and attention (2). Secondary outcomes included adverse events (safety analysis), change in brain iron burden measured by quantitative susceptibility mapping MRI (target engagement), and brain volume changes (secondary efficacy measure).

**Results:**

81 participants were recruited and randomized in a 2:1 ratio (53 drug:28 placebo). Baseline characteristics were equivalent between arms. 54 participants completed the study (withdrawals = 7 placebo and 20 deferiprone arm). Quantitative susceptibility mapping of the hippocampus at baseline and 12 months confirmed a significant (β[95%CI]:‐6.7 [‐11.0,‐2.5]; P = 0.004) lowering of brain iron in the deferiprone arm (mean change:‐3.71 ppb, 95% CI:‐7.24, ‐0.18) compared to placebo, whose iron levels increased during this timeframe (+3.03 ppb, 95%CI: 0.27, 5.80). Participants in the Deferiprone arm showed large statistically significant cognitive decline (β[95%CI]:‐0.295 [‐0.456, ‐0.135], P<0.001, Cohen’s d: ‐0.704) compared to placebo (deferiprone mean change, [95%CI]:‐0.86, [‐1.12, ‐0.61]; placebo mean change, [95%CI]:‐0.27, [‐0.55, 0.00]).

**Conclusion:**

Deferiprone (30 mg/kg/day) treatment markedly accelerated cognitive decline in people with amyloid‐confirmed MCI and mild AD. These findings highlight the importance of iron for cognition in AD, and provoke new questions. It is possible that iron elevation in AD is protective, or represents iron being sequestered inappropriately (e.g. deposited in pathology) leading to functional iron deficiency; or the chosen dose of deferiprone was too high for this condition.

